# The Cost of Male Aggression and Polygyny in California Sea Lions (*Zalophus californianus*)

**DOI:** 10.1371/journal.pone.0012230

**Published:** 2010-08-17

**Authors:** Leah R. Gerber, Manuela González-Suárez, Claudia J. Hernández-Camacho, Julie K. Young, John L. Sabo

**Affiliations:** 1 School of Life Sciences, Arizona State University, Tempe, Arizona, United States of America; 2 Laboratoire d'Ecologie et Evolution CNRS - UMR 7625, Université Pierre et Marie Curie, Paris, France; University of Pretoria, South Africa

## Abstract

In polygynous mating systems, males often increase their fecundity via aggressive defense of mates and/or resources necessary for successful mating. Here we show that both male and female reproductive behavior during the breeding season (June–August) affect female fecundity, a vital rate that is an important determinant of population growth rate and viability. By using 4 years of data on behavior and demography of California sea lions (*Zalophus californianus*), we found that male behavior and spatial dynamics—aggression and territory size—are significantly related to female fecundity. Higher rates of male aggression and larger territory sizes were associated with lower estimates of female fecundity within the same year. Female aggression was significantly and positively related to fecundity both within the same year as the behavior was measured and in the following year. These results indicate that while male aggression and defense of territories may increase male fecundity, such interactions may cause a reduction in the overall population growth rate by lowering female fecundity. Females may attempt to offset male-related reductions in female fecundity by increasing their own aggression—perhaps to defend pups from incidental injury or mortality. Thus in polygynous mating systems, male aggression may increase male fitness at the cost of female fitness and overall population viability.

## Introduction

In species with polygynous mating systems, males increase their reproductive success (fecundity) by either defending females (female-defense polygyny), defending key resources required by females for reproduction, such as breeding or nesting sites (resource-defense polygyny), or by establishing male dominance hierarchies that influence female choice of males (lek polygyny; [1], [2], [3]). In spite of literature demonstrating male fecundity (i.e., the number of offspring sired by a male) increases with polygyny (e.g., [4], [5], [6], [7], [8], [9], [10]), little empirical work has addressed the impact of aggressive behaviors often associated with polygyny on female fecundity. Specifically, interactions between males to secure resources or mates during the breeding season could have a detrimental effect on the ability of females to care for offspring (e.g., [11]). Infanticide and accidental mortality of offspring resulting from male-male combat are extreme examples of this [12], [13], [14], [15], [16], [17], [18]. Even fewer studies have explored the population consequences of male-male interactions in polygynous mating systems. Demography is determined in large part by female vital rates [19] although many studies have illustrated the importance of male vital rates [20], [21], [22], [23], [24]. Thus, any reduction in female fecundity due to male-male interactions associated with mate or breeding site defense could reduce the overall population growth rate.

In this paper, we explore the relationship between behavior and female fecundity in California sea lions (*Zalophus californianus*) within the Gulf of California, Mexico, with particular attention to the effect of intra-sexual aggressive behavior on the long term population growth rate of this population. Sea lions are polygynous mammals that aggregate at terrestrial breeding sites during the summer reproductive season [25], making them ideal for studies of behavior and demography [26]. Males do not participate in parental care; rather males increase their reproductive success by having multiple mates. Thus, male reproductive success may be related to agonistic interactions of territorial males and indirectly related to female future reproductive success. Unlike males, females actively participate in parental care [27]. Females alternate between foraging trips at sea (3–4 days) and nursing periods on land to support themselves and their pups during the ^∼^ one-year lactation period [28]. Pup survival depends primarily on the mother's ability to obtain sufficient food and provide protection against conspecific aggression [29], [30].

California sea lions are thought to exhibit a resource-defense polygynous breeding system, although some characteristics of their mating systems suggest it is lek-like [31]. During the breeding season, reproductively active males defend territories that vary in size, density of females, and topography (e.g. sandy vs. rocky beaches) [27], [32]. Aggressive encounters between males are frequent and males who initiate interactions (likely the more aggressive males) are also the ones who hold territories [33]. Females compete with other females to maintain space required for thermoregulation and pup care [31]. More aggressive females tend to have a competitive advantage when colony density is high [34]. Finally, while females typically defend their pups against conspecific attacks and accidental trampling by males engaged in aggressive territorial behavior, they must spend a significant amount of time at sea to eat. Pups are left on land and likely at higher risk of mortality when mothers are at sea foraging.

Here we examine how behaviors such as maternal attendance, male and female aggression, and spatial variables such as male territory size relate to female fecundity and thus, the population growth rate of California sea lions in the Gulf of California, Mexico [26]. Male aggression should simultaneously reduce time available for copulation with females and potentially increase the risk of trampling of pups. By contrast, female aggression should lend to better protection of pups from trampling (by males) and from aggressive females, as well as better defense of prime breeding grounds coveted by other females attending pups. Thus, we expect that female and male aggression may have countervailing effects on female fecundity and fecundity will decline with male aggression but increase with female aggression. We explore these predictions at the population-level using data for 6 island breeding colonies in the Gulf of California.

## Results

Of our putative predictor variables, only two were found to be correlated: the number of females per territory and territory size (Pearson's correlation, *r* = 0.428; *P* = 0.0003; Table 1). We considered territory size in subsequent analyses because this variable is a comprehensive predictor representing both space use and male defense of females.

**Table 1:** Pearson correlation among seven behavioral and spatial variables recorded at six California sea lion breeding colonies in the Gulf of California.

Variables^a^	*F*	*M*	*N*	*P*	*D*	*NF*
*F*						
*M*	0.082					
*N*	0.149	0.065				
*P*	−0.001	−0.030	0.024			
*D*	−0.255	−0.323	−0.315	0.182		
*NF*	0.052	−0.262	−0.215	−0.187	0.270	
*T*	−0.003	−0.003	−0.128	0.078	0.013	0.428*

*Significant correlation, *P* = 0.0003.

a *F* = Female aggression, *M* = Male aggression, *N* = Female nursing, *T* = Male Territory size, *P* = Male patrolling, D = Distance to nearest neighbor (among territorial males), *NF* = number of females in territory. Descriptions are provided in Table S4.

Model selection results suggested that the best multiple regression model for fecundity included 3 variables: female aggression, male aggression, and male territory size (Tables 2 & S1). This model had a reasonable fit as well (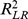 = 0.25; see Table 2). Four other models also had ΔAIC_C_ values of <2. Female and male aggression and territory size were included in 3 of the 4 supported models (Table S1). The best regression model for prospective fecundity included only 1 variable — female aggression — but this variable explained very little of the variance (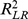 = 0.04). The model including female aggression as the single predictor garnered strong support (*w_i_* = 0.48) and was the only model with ΔAIC_C_<2 (Tables 3 & S2).

**Table 2:** Top candidate models explaining fecundity in California sea lions.

Model^a^	*Coefficient estimates*							*w*_i_	*R^2^_LR_*	Variable weights^b^	AIC_C_
	Intercept	*D*	*F*	*M*	*N*	*P*	*T*				
FMT*	**1.277**	**–**	**0.074**	**−0.170**	**–**	**–**	**−0.001**	**0.154**	0.254	−	25.3
FM*	1.178	–	0.044	−0.177	–	–	–	0.120	0.065	−	25.8
P*	0.794	–	–	–	–	0.612	–	0.103	0.026	0.27	26.1
T*	1.275	–	–	–	–	–	−0.001	0.080	0.024	**0.403**	26.6
D*	1.180	−0.023	–	–	–	–	–	0.073	0.021	0.297	26.8
*N*	1.139	–	–	–	−0.477	–	–	0.046	0.014	0.173	27.7
*FMD*	1.210	−0.025	0.048	−0.167	–	–	–	0.046	0.103	−	27.7
*F*	1.095	–	0.004	–	–	–	–	0.044	0.014	**0.557**	27.8
*M*	1.129	–	–	−0.007	–	–	–	0.044	0.014	**0.561**	27.8

Parameter estimates, Akaike weights (*w*_i_), and estimate of model fit (*R^2^_LR_*) for the top 9 candidate models explaining fecundity (i.e., the same year behavior was measured). We include all models with ΔAIC_C_<3 here so that we can calculate variable weights for all six variables considered (see Table 3). Weights for coefficients included in the model with the most AIC support and estimates for these coefficients are in bold.

a *F* = Female aggression, *M* = Male aggression, *T* = Male Territory size, *P* = Male patrolling, *D* = Distance to nearest neighbor (among territorial males), *N* = Female nursing behavior. Descriptions are provided in Table S4.

b Variable weights are the sum of weights of all models in the set considered containing that variable. * Indicates models with strong support (e.g., with ΔAIC_C_<2).

**Table 3:** Parameter estimates for the best candidate models predicting fecundity (the same year behaviors were measured) and prospective fecundity (the year following behavioral observations) in California sea lions.

Parameter	Estimate^a^	Se	Weighted Estimate^b^	*F*	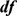	*P*
Fecundity the same year behavior was measured						
Intercept	1.277	0.156	1.146	–	–	–
*F*	0.074	0.020	0.027	13.020	1, 21.3	0.002
*M*	−0.170	0.038	−0.078	21.080	1, 16.8	0.000
*T*	−0.001	0.0004	0.0002	5.750	1, 20.8	0.026
Prospective fecundity: fecundity the next year behavior was measured						
Intercept	0.356	0.236	–	–	–	–
*F*	0.105	0.038	–	7.54	1, 11.6	0.018

*F* = Female aggression, *M* = Male aggression, *T* = Male Territory size.

a Estimates of parameters from candidate models with highest support (highest Akaike weight).

b Weighted estimates are calculated as in Burnham and Anderson [66].

**Figure 1:**
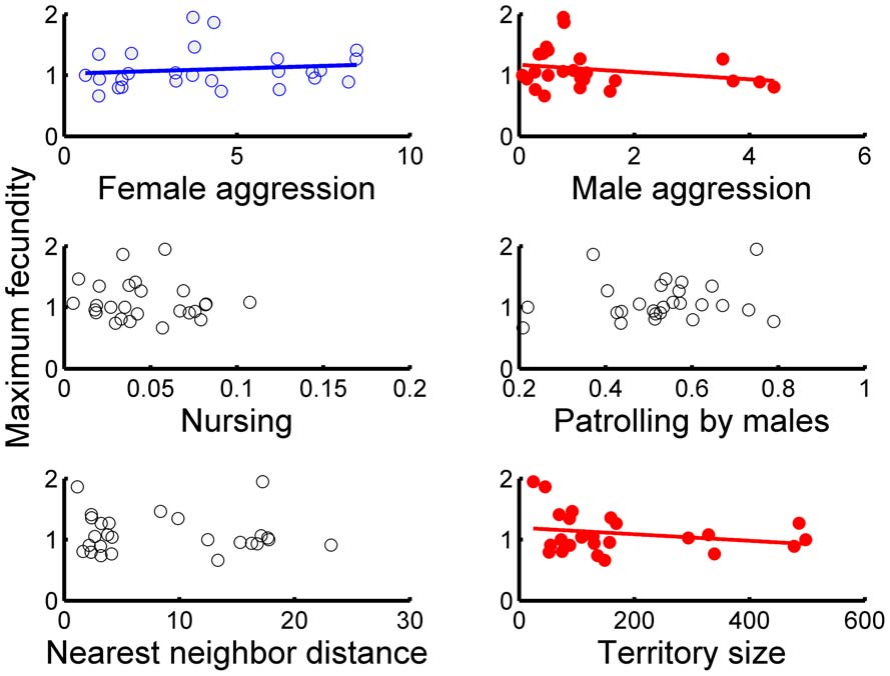
Univariate relationships between the six candidate behavioral variables and fecundity (e.g., female fecundity the same year behaviors were observed). Fitted lines are significant where present.

**Figure 2:**
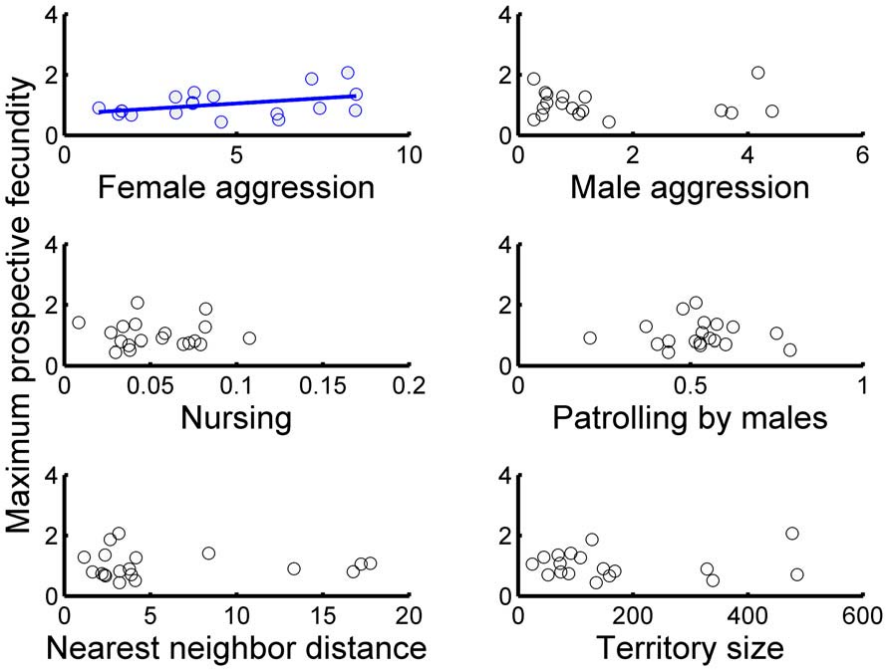
Univariate relationships between the six candidate behavioral variables and prospective fecundity (e.g., female fecundity the year after behaviors were observed). Fitted lines are significant where present.

Female aggression, male aggression, and male territory size exerted significant linear effects on fecundity but the direction of these effects varied (Fig. 1; Table 3). Female aggression had positive effects on fecundity, whereas male aggression had negative effects on fecundity (Table 3). Male territory size was also negatively associated with fecundity, but the effect size was small. Finally, female aggression was positively related to prospective fecundity (Fig. 2; Table 3).

## Discussion

Polygyny in California sea lions most likely increases the average fecundity of territorial males as well as the variance in the fecundity of all males, such that there is greater disparity in fecundity between territorial and non-territorial males. In this paper we provide evidence that male characteristics associated with polygyny have potentially detrimental effects on female fecundity. Specifically, we found significant negative effects of both male aggression and increased territory size on female fecundity. By contrast, we found significant positive effects of female aggression on this estimate of female fecundity. Of six variables examined (see Tables 1 & 2), male aggression, male territory size, and female aggression explained ∼25% of the variance in female fecundity and male variables comprised >65% of variable weights across all regression models examined. By contrast, only female aggression was significantly related to prospective fecundity.

Our observed negative relationship between male aggression and female fecundity is consistent with observations of common lizards (*Zootoca vivipara*; [22]) and Hawaiian monk seals (*Monachus schauinsland*; [35]). These results suggest several interesting hypotheses about the relationship between aggression and fecundity in sea lions. Male aggression may have negative effects on female fecundity by directly influencing female behavior and female-pup interactions. For example, male aggression may cause separation of mother-pup pairs, increase female vigilance and disrupt nursing which could make pups more susceptible to external sources of mortality [36], disrupt resting in females and pups, and possibly cause direct mortality of pups—trampling by large males during territorial bouts. Alternatively, male aggression may reduce female fecundity indirectly due to a male trade-off between time spent on courtship (and copulation) and defense of a territory leading to a lower insemination rate of philopatric females [37]. Reproductive effort in male red deer (*Cervus elaphus*) decreases with increasing density [38], [39] because males increase the frequency of fighting with increasing density [40]. Similarly, male domestic cats (*Felis catus*) that are more aggressive have reduced male fecundity both directly due to their propensity for fighting in lieu of mating and indirectly by scaring off females [41], [42], [43]. While we did not identify a significant relationship between male aggression and prospective fecundity, these two hypotheses deserve further exploration to unravel the mechanisms by which male aggression reduces fecundity.

We found that female aggression is significantly and positively related to female fecundity (the same year) and prospective fecundity (next year). Interestingly, in other species, female aggression has been linked to a decrease in fecundity. For example, Harcourt [30] found that aggression was correlated with pup mortality in a dense population of South American fur seal (*Arctocephalus australis*). Similarly, female reproductive success is enhanced through an increase in pup survival when aggression by female elephant seals to other females and alien pups is infrequent [44]. Our results suggest that present levels of female aggression in California sea lions may instead be associated with increased fecundity rates. Female aggression may result in higher fecundity by increasing survival of pups directly via greater protection of the pups or indirectly by securing higher quality resting and breeding areas within the colony for females and their pups [45]. Females that are more fit may invest more time in fighting to delineate a space for themselves and pups [17], [30]. Female grey seals (*Halichoerus grypus*) are known to aggressively defend space for themselves and their pups [46]. Therefore, female aggression may represent a form of vigilance, where aggressive females are exhibiting higher rates of maternal care. Vigilant parenting may also explain the relationship between female aggression and prospective fecundity of philopatric females. Alternatively, increased female aggression may be the outcome of higher fecundity, as females with pups tend to be more aggressive than single females [47]. Future studies should attempt to determine the mechanisms of this relationship and also measure the timing (as opposed to frequency) of male and female aggression.

Lower fecundity was also associated with larger territory sizes, although the magnitude of the effect was quite small (Table 3). In the colonies we studied, larger territories are often found in colonies with lower densities and declining population trends (e.g., Granito, see [32]). Therefore, larger territories in our study sites may simply reflect lower population densities (less competing males) and possible lower quality sites (where fewer males try to set a territory and individuals are less fit). Both lower densities and lower quality habitat could reduce fecundity rates explaining the association between territory size and fecundity we observed. Studies that explicitly consider habitat quality and fecundity are needed to explore these hypotheses.

We used simple linear models to explore relationships between behavior and demography (fecundity) thereby potentially ignoring possible non-linearities in these relationships. The decision to use linear models was made because the sample size requirements of non-linear models are higher than the number of observations in our dataset. Moreover, an initial visual inspection of our data did not suggest any obvious non-linearities in any of the univariate response surfaces. However, we recognize that there are reasons to expect the relationship between somes behaviors and fecundity to be non-linear. For example, female aggression is likely to have a non-linear relationship with fecundity because low to intermediate levels of female aggression may help secure suitable habitat and protect pups (as suggested by our study), while high levels of female aggression disrupt maternal care reducing the time females devote to nurse their pups and also place pups at higher risk of injury [30]. Future studies—with higher sample size and wider ranges of variation in behavior—should attempt to construct and test nonlinear hypotheses using more data-intensive tools than used here (e.g. Generalized Additive Models [48]).

Overall, our results indicate that female and male behavior influence female fecundity in sea lions. However, measured behavior explained only 25% of the variability in fecundity in our study sites. Female fecundity is likely influenced by other factors not measured in our study, such as habitat quality and food abundance (e.g., [49]). Furthermore, an alternative mating strategy might exist that is linked to female fecundity (i.e.; the existence of males that fertilize females without venturing ashore during the breeding season, or perhaps do so at night, outside of the observation periods in the study; [50], [51], [52], [53]). In this case, female fecundity would not be explained by territorial male behavior on site. Future studies should consider the effect of these factors in sea lion female fecundity and also explore how different conditions may influence behavioral responses. For example, aggression patterns in California sea lions have been shown to be influenced by habitat conditions such as temperature and sea condition [54] and extrinsic factors have been suggested to best explain population dynamics of elephant seals [55]. Nevertheless, our study provides an initial understanding of the complexity of factors that affect vital rates such as fecundity, and reveals the importance of considering animal behavior in studies of population dynamics.

Our observation that female fecundity varies with male aggression has important implications for understanding the relationship between polygyny and demography. Specifically, female fecundity is an important parameter in estimating the growth rate and viability of California sea lions. Small changes in fecundity may represent a significant influence on the discrete annual rate of population increase (λ). For example, we observed values for the number of pups per female ranging from 0.44–2.07. Applying extreme values from this range (2.1 vs. 0.4) into a projection matrix based on measured survival rates (see [26] for details) we estimated changes in the annual rate of increase of −0.04 (from 1.16 to 1.12). This small change in λ represents a 4% reduction in the annual population growth of this sea lion population. Thus, our observation that female fecundity varies with aggression (by both sexes) and male territory size suggests that female and male social interactions may influence the propensity for populations to recover from decline or increase their risk of extinction (e.g., [21], [35]).

In conclusion, our data illustrate the importance of considering male dynamics in population models typically used in conservation. Male behavior has a much stronger relationship with female vital rates than similar female behaviors during the same year. For example, the proportional contribution of weights of male coefficients (*M* & *T*: 0.43; Table 2) was >1.5 fold higher than the proportion of variable weights associated with single female coefficient from best model (*F*: 0.25; Table 2). Males matter, in this case, not because male limitation precludes efficient siring of offspring [21], but because high aggression among males apparently suppresses reproduction by females. At the same time, female aggressive behavior explains some variation in fecundity in subsequent years. Studies such as ours that examine the mechanisms that determine demography (e.g., behavior) may inform practical decision making for imperiled populations by identifying mechanisms that are most critical to population viability [56]. Understanding which behavioral and ecological factors influence population dynamics can encourage proactive management and better predictions of wildlife response to changes. For example, increases in male aggression were associated with reduced female fecundity, suggesting that environmental changes that may increase male aggression (e.g., greater human disturbance, increases in density) are likely to be associated with reduced fecundity.

## Materials and Methods

### Data Collection

**Figure 3:**
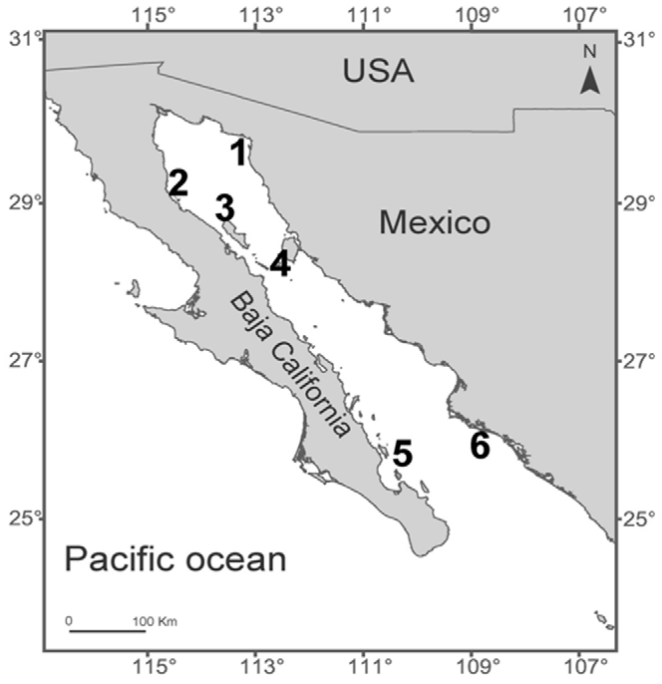
Study sites in the Gulf of California, Mexico. Numbers indicate the six islands where observations were conducted: (1) San Jorge, (2) Los Lobos, (3) Granito, (4) San Esteban, (5) Los Islotes, and (6) Farallon de San Ignacio.

Our analyses stem from 4 years of spatially replicated observations at 6 breeding colonies in the Gulf of California, Mexico (Fig. 3). All field research was approved by the Institutional Animal Care and Use Committee at Arizona State University (permit 07-918R). Observations were collected simultaneously at 2 sites per island during July 2004–2006 (behavioral data) and July 2004–2007 (demographic data, Table S3). During each observation trip, we gathered data for 8–9 hrs per day, but the actual times of observation varied daily to ensure our observations covered all daylight hours (7 am–7 pm). We concentrated our efforts in July because at this time most births have already occurred and mating has started [28], [31]. García-Aguilar and Aurioles-Gamboa [28] found that the reproductive peak occurs approximately at the same time at Granito and Los Islotes, although there appears to be two peaks at Los Islotes. These data suggest that differences in reproductive timing among islands are unlikely to introduce bias to our study design.

We recorded data on 4 behaviors associated with mating and reproductive success: female aggression, male aggression, nursing (in females), and patrolling (in territorial males; see Table S4 for definitions). For aggression, we observed all male and female sea lions present at the study sites. Repeated aggressive interactions between two individuals were treated as a single event if they occurred ≤3 min of one another. Interactions initiated >3 min after the last bout ended were recorded as a new, independent event [33], [54], [57], [58]. Nursing and patrolling behaviors were obtained from scan sampling (Table S4). Scan samples document the behavior of each animal when it is first observed [59]. The behavior and sex of each adult at the study site was recorded during each scan.

Each day we also documented 3 territorial attributes that describe spatial dynamics of individually-identified territorial males: distance to nearest neighbor, territory size, and number of females per territory (see Table S4 for definitions). Additional details on the size of colonies, sex ratio, and density are provided in [26], [33], [54]. For all behaviors and territorial attributes, we calculated a single value per year as the mean of all recorded observations during the observation trips (Table S3).

Individual females could not be identified, thus we do not examine behavior at the level of the individual, but as population averages. Unlike females, male sea lions have distinct scar patterns, generally permitting individual identification. In a few cases (∼7%) we were unable to identify individual males, thus some could be overrepresented in our sample as they could be observed many times per day, trip, or year. In a previous study [54] we explored this potential bias with a subset of identified males and found that individuals were generally represented equally in our estimates of aggressive behavior, suggesting our data represented the overall population rather than a few particularly aggressive individuals.

We also estimated a single value per year for female fecundity which was calculated as the ratio of the maximum number of pups to the maximum number of adult females observed at each site. We calculated fecundity the same year the behavior was recorded (hereafter, fecundity) and fecundity the year after behavior was recorded (hereafter, prospective fecundity). Counts were completed 4–6 times a day at each site during observation trips (Table S3). During each count observers are unlikely to detect all individuals in the colony, especially females and pups. Pups may rest amongst or under large rocks and boulders and thus, are undetected by observers. Similarly, some females are likely to be foraging at sea during any given count. Previous research suggests that up to 50% of pups and 23–54% of females can be undetected in population counts [60], [61]. In the Gulf of California, the proportion of missing animals is thought to be consistent among island study sites (Stzeren et al. 2006). To account for the likely underestimates in abundance from raw counts, we use the maximum (rather than mean) number of individuals observed in each case as the maximum is most likely a better estimate of the actual number of adult females and pups (leading to more accurate estimates of fecundity). However, using the maximum value could inflate the variance and lower the precision of our estimates. To explore this potential source of error, we tested for differences in the variance of maximum and mean fecundity measures, where mean fecundity was calculated as the ratio of the mean number of pups to mean number of females observed per year at each site (Table S5). We found no significant differences (*F* = 0.59, *n* = 25, 25, *P* = 0.9; *F* = 1.24, *n* = 15, 15, *P* = 0.33; current and prospective fecundity, respectively; Table S5). Therefore, our use of the maximum values in the calculation of the ratio of pups: females should not inflate the variance or lower the precision of our estimates of this parameter.

To ensure that our estimates of fecundity are realistic, we compare them with published fecundity rates obtained from mark-recaptured studies completed at one of the studied colonies for females 10–25 year olds (Los Islotes, [57]) and the California Channel Islands for females 5–10 years old [62]. The mean birth rates weighted by the sample size were 0.54 for Hernandez-Camacho et al. [57] and 0.69 for Melin [62]. These values correspond well with our mean estimate of 0.70 for Los Islotes (Table S5), suggesting that our estimates based on count data are likely to accurately reflect fecundity rates.

### Data Analysis

We identified relevant behavioral and spatial variables that affect fecundity rates using multivariate linear regression models. To avoid co-linearity in our models we identified correlations among the behavioral and spatial variables described above (Table S4) using pairwise Pearson correlations. To be conservative, variables with *r*>0.4 were considered to be correlated. All uncorrelated variables were then used to generate linear regression models. These models were used to predict fecundity the same year the behavior was recorded and prospective fecundity (fecundity the year after behavior was recorded). For 2 of our 6 surveyed islands, we had only 1 year of data (Table S3). Thus, we used data from 6 islands for our analysis of fecundity, but only 4 islands for our analysis of prospective fecundity.

We fitted regression models using PROC MIXED in SAS [63] and an automated macro implementing all-possible model selection [64]. Our regression models considered year as a repeated measure with a first order autoregressive variance structure with sites (2 per island) as subjects, nested within island. This approach accounts for spatial and temporal variation while analyzing fixed effects of behavior and spatial variables on female fecundity. In our analysis, the unit of replication is the site (2 per island) for which there are repeat measurements (across years, not days) of behavioral variables and maximum fecundity estimates. Repeat measurements introduce statistical dependence in the variance structure, and our analysis takes this source of statistical dependence into consideration by modeling the site as a subject that is measured repeatedly. In other words, we use a repeated measures design in our regression models that assumes strong temporal autoregressive dependence (AR1 variance structure). This analysis corrects for statistical dependencies among repeat observations of the same sites over time. Although only 3 islands were surveyed on all 4 years (Table S3), the unbalanced nature of the data was not an issue for our approach because we estimated model parameters via maximum likelihood rather than least squared methods, the former not requiring a balanced design in time.

We used Akaike's Information Criterion corrected for small sample sizes (AIC_C_) to choose among all possible candidate models [65], [66]. Due to low sample size (*n* = 26, *n* = 19 for fecundity and prospective fecundity, respectively), we considered only candidate models with ≤3 predictor variables (i.e., *k* = 9, including only 3 behaviors). This maximized the ratio of data to explanatory variables in fitting our behavioral models to fecundity data (*n/k* = 2–3). All candidate models with ΔAIC_C_<2 were considered supported models [66]. If ≥1 model was supported, the average parameter estimates were calculated as described by Burnham and Anderson [66]. Although standard estimates of model fit (*R^2^*) are not available for mixed models, we calculated the likelihood ratio *R^2^* (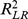) which provides a useful estimate of the proportion of variance explained by a mixed model [67].

### Methodological caveats

We note that our analysis of relationships between behaviors and fecundity should be viewed with some caution. First, our analyses push the limit of acceptable ratios of information (sample size) to structural parameters in our statistical model. Our models included up to 3 behaviors, time as a repeated measure, several spatial factors (site and island), and a covariance parameter associated with the variance structure in our repeated measures analysis. Thus, our ratio of data to variables (*k* = 9 including the intercept and MSE) was ca. 2.1–2.9. Burnham and Anderson [66] advise an *n/k* ratio of ∼10 for noisy data, but also suggest that small sample size corrections in the AIC_c_ calculation will correctly adjust for small sample size and high numbers of predictors. Our decision to analyze only subsets of ≤3 predictors represents a compromise between a strict interpretation of Burnham and Anderson's rule of thumb, and much lower *n*/*k* ratios of models including all of our uncorrelated behaviors. Nevertheless, our application of AIC_C_, may correctly adjust AIC values for small sample size bias, but should not be interpreted to completely preclude the possibility of our large model overfitting a relatively small dataset. Second, model selection in multiple regression involving both fixed and random factors is not well understood; it is still largely unknown how best to conduct model selection in mixed effects models (e.g., whether AIC_C_, BIC or other information criteria are best suited). Finally, we recognize that it is impossible to distinguish correlation from causation in our identified relationships between fecundity and behavior.

## Supporting Information

Table S1 (0.06 MB DOC)

Table S2 (0.06 MB DOC)

Table S3 (0.03 MB DOC)

Table S4 (0.03 MB DOC)

Table S5 (0.07 MB DOC)
