# Supplementary material for: The Cost of Male Aggression and Polygyny in California Sea Lions (Zalophus californianus)
Source: PLoS One. 2010 Aug 17;5(8):e12230. doi: 10.1371/journal.pone.0012230 (PMC2923196; doi:10.1371/journal.pone.0012230)
Supplement: Table S1 — (0.06 MB DOC) [file pone.0012230.s001.doc]

Table S1: Model selection results from an all possible subsets (of all combinations of 3 fixed behavioral effects) of multiple regression relating behaviors (defined below) to fecundity (i.e., the same year behaviors were measured) in California sea lions. Model parameter abbreviations are female aggression (*F*), male aggression (*M*), male territory size (*T*), male patrolling (*P*), distance to nearest neighbor among territorial males (*D*), and nursing by females (*N*). Each model is ranked by its Akaike Information Criteria value (corrected for small sample size, AIC_C_) and associated ΔAIC_C_ (ΔAIC_C_ = 0 is the best model).

| Model | *k*^a^ | AIC_C_ | *N* | ΔAIC_C_ | *w*_i_ |
| --- | --- | --- | --- | --- | --- |
| *FMT* | 3 | 25.3 | 25 | 0 | 0.15 |
| *FM* | 2 | 25.8 | 25 | 0.5 | 0.12 |
| *P* | 1 | 26.1 | 26 | 0.8 | 0.10 |
| *T* | 1 | 26.6 | 25 | 1.3 | 0.08 |
| *D* | 1 | 26.8 | 26 | 1.5 | 0.07 |
| *N* | 1 | 27.7 | 25 | 2.4 | 0.05 |
| *FMD* | 3 | 27.7 | 26 | 2.4 | 0.05 |
| *F* | 1 | 27.8 | 25 | 2.5 | 0.04 |
| *M* | 1 | 27.8 | 26 | 2.5 | 0.04 |
| *PD* | 2 | 28.6 | 26 | 3.3 | 0.03 |
| *DT* | 2 | 29.3 | 25 | 4 | 0.02 |
| *FMP* | 3 | 29.4 | 26 | 4.1 | 0.02 |
| *FMN* | 3 | 29.5 | 26 | 4.2 | 0.02 |
| *PT* | 2 | 29.7 | 25 | 4.4 | 0.02 |
| *FP* | 2 | 29.8 | 26 | 4.5 | 0.02 |
| *MN* | 2 | 29.9 | 26 | 4.6 | 0.02 |
| *MP* | 2 | 29.9 | 26 | 4.6 | 0.02 |
| *NP* | 2 | 29.9 | 26 | 4.6 | 0.02 |
| *FD* | 2 | 30.2 | 25 | 4.9 | 0.01 |
| *FT* | 2 | 30.4 | 25 | 5.1 | 0.01 |
| *MT* | 2 | 30.4 | 25 | 5.1 | 0.01 |
| *NT* | 2 | 30.4 | 25 | 5.1 | 0.01 |
| *MD* | 2 | 30.5 | 25 | 5.2 | 0.01 |
| *ND* | 2 | 30.5 | 26 | 5.2 | 0.01 |
| *FN* | 2 | 31.5 | 26 | 6.2 | 0.01 |
| *PDT* | 3 | 32.6 | 25 | 7.3 | 0.00 |
| *FPD* | 3 | 32.8 | 26 | 7.5 | 0.00 |
| *MPD* | 3 | 32.8 | 26 | 7.5 | 0.00 |
| *NPD* | 3 | 32.8 | 26 | 7.5 | 0.00 |
| *FDT* | 3 | 32.9 | 25 | 7.6 | 0.00 |
| *MND* | 3 | 33.5 | 25 | 8.2 | 0.00 |
| *NDT* | 3 | 33.6 | 25 | 8.3 | 0.00 |
| *MDT* | 3 | 33.7 | 25 | 8.4 | 0.00 |
| *FNP* | 3 | 34 | 26 | 8.7 | 0.00 |
| *NPT* | 3 | 34 | 25 | 8.7 | 0.00 |
| *FPT* | 3 | 34.1 | 25 | 8.8 | 0.00 |
| *MNP* | 3 | 34.1 | 25 | 8.8 | 0.00 |
| *MPT* | 3 | 34.1 | 25 | 8.8 | 0.00 |
| *FND* | 3 | 34.4 | 26 | 9.1 | 0.00 |
| *FNT* | 3 | 34.7 | 25 | 9.4 | 0.00 |
| *MNT* | 3 | 34.8 | 25 | 9.5 | 0.00 |

^a^Number of behavioral variables included in model, actual number of parameters is *k* + 6 including time, site, island and the covariance parameter included in first order auto-regressive variance structure of repeated measure.
