# Supplementary material for: The Cost of Male Aggression and Polygyny in California Sea Lions (Zalophus californianus)
Source: PLoS One. 2010 Aug 17;5(8):e12230. doi: 10.1371/journal.pone.0012230 (PMC2923196; doi:10.1371/journal.pone.0012230)
Supplement: Table S2 — (0.06 MB DOC) [file pone.0012230.s002.doc]

Table S2: Model selection results from an all possible subsets (of all combinations of 3 fixed behavioral effects) of multiple regression relating behaviors (defined below) to prospective fecundity (i.e., fecundity the year after behaviors were measured) in California sea lions. Model parameter abbreviations are female aggression (*F*), male aggression (*M*), male territory size (*T*), male patrolling (*P*), distance to nearest neighbor among territorial males (*D*), and nursing by females (*N*). Each model is ranked by its Akaike Information Criteria value (corrected for small sample size, AIC_C_) and associated ΔAIC_C_ (ΔAIC_C_ = 0 is the best model).

| Model | *k*^a^ | AIC_C_ | *N* | ΔAIC_C_ | *w*_i_ |
| --- | --- | --- | --- | --- | --- |
| *F* | 1 | 33 | 19 | 0 | 0.48 |
| *FD* | 2 | 36.2 | 19 | 3.2 | 0.10 |
| *D* | 1 | 37.5 | 19 | 4.5 | 0.05 |
| *FP* | 2 | 37.5 | 19 | 4.5 | 0.05 |
| *N* | 1 | 37.7 | 19 | 4.7 | 0.05 |
| *M* | 1 | 37.9 | 19 | 4.9 | 0.04 |
| *T* | 1 | 37.9 | 19 | 4.9 | 0.04 |
| *P* | 1 | 38 | 19 | 5 | 0.04 |
| *FN* | 2 | 38.1 | 19 | 5.1 | 0.04 |
| *FM* | 2 | 38.2 | 19 | 5.2 | 0.04 |
| *FT* | 2 | 39.2 | 18 | 6.2 | 0.02 |
| *FPD* | 3 | 42.1 | 19 | 9.1 | 0.01 |
| *MD* | 2 | 42.3 | 19 | 9.3 | 0.00 |
| *FMD* | 3 | 42.4 | 19 | 9.4 | 0.00 |
| *FND* | 3 | 42.4 | 19 | 9.4 | 0.00 |
| *ND* | 2 | 42.5 | 19 | 9.5 | 0.00 |
| *NP* | 2 | 42.5 | 19 | 9.5 | 0.00 |
| *DT* | 2 | 42.6 | 18 | 9.6 | 0.00 |
| *MP* | 2 | 42.6 | 19 | 9.6 | 0.00 |
| *PD* | 2 | 42.6 | 19 | 9.6 | 0.00 |
| *MN* | 2 | 42.7 | 19 | 9.7 | 0.00 |
| *NT* | 2 | 42.7 | 18 | 9.7 | 0.00 |
| *MT* | 2 | 43.2 | 18 | 10.2 | 0.00 |
| *PT* | 2 | 43.2 | 18 | 10.2 | 0.00 |
| *FNP* | 3 | 43.5 | 19 | 10.5 | 0.00 |
| *FMP* | 3 | 43.7 | 19 | 10.7 | 0.00 |
| *FDT* | 3 | 44.3 | 18 | 11.3 | 0.00 |
| *FMN* | 3 | 44.3 | 19 | 11.3 | 0.00 |
| *FPT* | 3 | 45 | 18 | 12 | 0.00 |
| *FMT* | 3 | 46 | 18 | 13 | 0.00 |
| *FNT* | 3 | 46 | 18 | 13 | 0.00 |
| *MPD* | 3 | 48.4 | 19 | 15.4 | 0.00 |
| *MND* | 3 | 48.5 | 19 | 15.5 | 0.00 |
| *MNP* | 3 | 48.5 | 19 | 15.5 | 0.00 |
| *NPD* | 3 | 48.5 | 19 | 15.5 | 0.00 |
| *NDT* | 3 | 49 | 18 | 16 | 0.00 |
| *MDT* | 3 | 49.2 | 18 | 16.2 | 0.00 |
| *NPT* | 3 | 49.2 | 18 | 16.2 | 0.00 |
| *MNT* | 3 | 49.4 | 18 | 16.4 | 0.00 |
| *PDT* | 3 | 49.4 | 18 | 16.4 | 0.00 |

^a^Number of behavioral variables included in model, actual number of parameters is *k* + 6 including year, site, island and the covariance parameter included in first order auto-regressive variance structure of repeated measure.
