# Supplementary material for: The Cost of Male Aggression and Polygyny in California Sea Lions (Zalophus californianus)
Source: PLoS One. 2010 Aug 17;5(8):e12230. doi: 10.1371/journal.pone.0012230 (PMC2923196; doi:10.1371/journal.pone.0012230)
Supplement: Table S3 — (0.03 MB DOC) [file pone.0012230.s003.doc]

**Table S3. Dates for field observation trips of California sea lion behavior on islands in the Gulf of California, Mexico.**

| **Island** | **2004** | **2005** | **2006** | **2007** |
| --- | --- | --- | --- | --- |
| San Jorge | 10-17 July | 15-20 July | 9-14 July | 22-27 July |
| Isla Lobos | 20-25 July | N/A | N/A | N/A |
| Granito | 21-26 July | 15-20 July | 9-14 July | 22-27 July |
| San Esteban | 10-17 July | N/A | 9-13 July | 22-26 July |
| Farallón de San Ignacio | 10-13,15-17 July | N/A | N/A | N/A |
| Los Islotes | 21-27 July | 21-24 July | 8-14 July | 22-28 July |
