# Supplementary material for: The Cost of Male Aggression and Polygyny in California Sea Lions (Zalophus californianus)
Source: PLoS One. 2010 Aug 17;5(8):e12230. doi: 10.1371/journal.pone.0012230 (PMC2923196; doi:10.1371/journal.pone.0012230)
Supplement: Table S4 — (0.03 MB DOC) [file pone.0012230.s004.doc]

**Table S4.** Ethogram and sampling description for field investigations on California sea lion behavior and demography.

|  | | Description | Sampling description |
| --- | --- | --- | --- |
| Behavior | |  |  |
| Aggression | | Open mouth display, vocalization, threat, grabbing, pushing and/or biting. Includes interactions with (e.g., biting) and without (e.g., vocalization) physical contact. | Mean hourly rate (total number of aggressions observed/observation effort) calculated from 1 hr observation bouts conducted 2-3 times a day during each field trip. |
| Nursing | | Female feeding pup (lactating). | Mean proportion of females nursing (females nursing/total females) obtained from scan samplings conducted 4-6 times per day during each field trip. |
| Patrolling | | Territorial male delimiting land or water territories by swimming or walking along the periphery of an area (i.e., territory) and repeatedly vocalizing. Males resting within the boundaries of a territory and repeatedly vocalizing were also considered to be patrolling. Territorial vocalizations were never directed to another individual in contrast to aggressive vocalizations (see above). | Mean proportion of males patrolling (males patrolling/total males) obtained from scan samplings conducted 4-6 times per day during each field trip. |
| Spatial dynamics (territorial attributes) | | |  |
| NN Distance (D) | Approximate distance in meters between a territorial male and the nearest of the territorial males present in the area. | | Mean distance calculated from 2-3 estimates recorded thorough the day during each field trip. Distance estimates were approximated in 5-meter interval ranges using a rangefinder and compass or a grid painted over the study site. |
| Territory size | Approximate area of the territory defended by a territorial male in m^2^. | | Mean territory size based on area estimates obtained 2-3 times a day during each field trip. Area estimates were calculated as the total terrestrial and/or aquatic surface patrolled and defended by a male (i.e., the area within the territory boundaries). Territory boundaries were defined from continuous observation. Generally areas were calculated with the assistance of a grid painted over the study site. |
| Females/territory | Number of females present in a specific territory. | | Mean number of females inside each territory estimated from 2-3 daily counts during each field trip. |
