# Supplementary material for: The Cost of Male Aggression and Polygyny in California Sea Lions (Zalophus californianus)
Source: PLoS One. 2010 Aug 17;5(8):e12230. doi: 10.1371/journal.pone.0012230 (PMC2923196; doi:10.1371/journal.pone.0012230)
Supplement: Table S5 — (0.07 MB DOC) [file pone.0012230.s005.doc]

**Table S5.** Estimates of maximum and mean fecundities per year and site. [[1]](#footnote-2)

| Year | Island | Site | Max | Mean^a^ | Stdev^b^ | N |
| --- | --- | --- | --- | --- | --- | --- |
| 2004 | San Jorge | 11 | 1.951 | 1.851 | 0.038 | 33 |
| 2004 | San Jorge | 12 | 1.462 | 1.562 | 0.027 | 36 |
| 2004 | Los Lobos | 21 | 1.027 | 0.727 | 0.034 | 36 |
| 2004 | Los Lobos | 22 | 1.346 | 1.022 | 0.031 | 32 |
| 2004 | San Esteban | 31 | 1.065 | 0.617 | 0.032 | 25 |
| 2004 | San Esteban | 32 | 0.955 | 0.822 | 0.024 | 31 |
| 2004 | Granito | 43 | 1.000 | 0.794 | 0.025 | 37 |
| 2004 | Granito | 44 | 0.913 | 0.712 | 0.021 | 23 |
| 2004 | Farallon | 51 | 1.000 | 1.084 | 0.029 | 31 |
| 2004 | Farallon | 52 | 0.939 | 0.253 | 0.015 | 48 |
| 2004 | Los Islotes | 61 | 0.662 | 0.669 | 0.011 | 43 |
| 2004 | Los Islotes | 62 | 0.932 | 0.937 | 0.022 | 40 |
| 2005 | San Jorge | 11 | 1.054 | 1.856 | 0.063 | 32 |
| 2005 | San Jorge | 12 | 1.410 | 1.317 | 0.019 | 36 |
| 2005 | Granito | 43 | 1.079 | 1.020 | 0.024 | 37 |
| 2005 | Granito | 44 | 1.038 | 1.056 | 0.061 | 19 |
| 2005 | Los Islotes | 61 | 0.906 | 0.942 | 0.017 | 62 |
| 2005 | Los Islotes | 62 | 0.806 | 0.612 | 0.028 | 37 |
| 2006 | San Jorge | 11 | 1.730 | 1.726 | 0.038 | 29 |
| 2006 | San Jorge | 12 | 1.231 | 1.163 | 0.023 | 42 |
| 2006 | San Esteban | 31 | 1.000 | 1.355 | 0.035 | 30 |
| 2006 | San Esteban | 32 | 0.698 | 0.828 | 0.021 | 30 |
| 2006 | Granito | 43 | 0.694 | 0.848 | 0.024 | 36 |
| 2006 | Granito | 44 | 1.190 | 0.984 | 0.019 | 35 |
| 2006 | Los Islotes | 61 | 0.592 | 0.584 | 0.016 | 24 |
| 2006 | Los Islotes | 62 | 0.676 | 0.433 | 0.024 | 39 |
| 2007 | San Jorge | 11 | 1.286 | 1.409 | 0.034 | 36 |
| 2007 | San Jorge | 12 | 0.667 | 0.717 | 0.018 | 36 |
| 2007 | San Esteban | 31 | 0.709 | 0.739 | 0.018 | 30 |
| 2007 | San Esteban | 32 | 0.517 | 0.762 | 0.025 | 31 |
| 2007 | Granito | 43 | 2.071 | 0.767 | 0.062 | 35 |
| 2007 | Granito | 44 | 0.818 | 0.657 | 0.017 | 35 |
| 2007 | Los Islotes | 61 | 0.445 | 0.651 | 0.011 | 34 |
| 2007 | Los Islotes | 62 | 0.700 | 0.777 | 0.027 | 39 |

^a^ Mean fecundity is estimated as the ratio of the mean number of pups to the mean number of females.

^b^ The standard deviation of this mean is approximated as , where $s_{F}^{2}$ and $s_{P}^{2}$ are the variance in the number of females and pups respectively.

1. Mean fecundity is estimated as the ratio of the mean number of pups to the mean number of females. The standard deviation of this mean is approximated as , where $s_{F}^{2}$ and $s_{P}^{2}$ are the variance in the number of females and pups respectively. [↑](#footnote-ref-2)
